# Evaluation of Low versus High Volume per Minute Displacement CO_2_ Methods of Euthanasia in the Induction and Duration of Panic-Associated Behavior and Physiology

**DOI:** 10.3390/ani6080045

**Published:** 2016-08-02

**Authors:** Debra L. Hickman, Stephanie D. Fitz, Cristian S. Bernabe, Izabela F. Caliman, Melissa M. Haulcomb, Lauren M. Federici, Anantha Shekhar, Philip L. Johnson

**Affiliations:** 1 Laboratory Animal Resource Center, Department of Cellular & Integrative Physiology, Indiana University School of Medicine, Indianapolis, IN 46202-5181, USA; 2 Department of Cellular & Integrative Physiology, Indiana University School of Medicine, Indianapolis, IN 46202, USA; 3 Medical Neuroscience Program at Stark Neurosciences Research Institute, Indiana University School of Medicine, Indianapolis, IN 46202, USA; cbernabe@iupui.edu (C.B.); lafederi@iupui.edu (L.F.); philjohn@iupui.edu (P.L.J.); 4 Department of Psychiatry, Indiana University School of Medicine, Indianapolis, IN 46202, USA; sdfritz@iupui.edu (S.D.F.); mhaulcom@iupui.edu (M.H.); ashekhar@iu.edu (A.S.); 5 Department of Anatomy & Cell Biology, Indiana University School of Medicine, Indianapolis, IN 46202, USA; icaliman@iupui.edu; 6 Indiana Clinical and Translational Sciences Institute, Indiana University School of Medicine, Indianapolis, IN 46202, USA

**Keywords:** CO_2_, hypercapnia, euthanasia, panic, anxiety, distress

## Abstract

**Simple Summary:**

Current recommendations for the use of CO_2_ as a euthanasia agent for rats require the use of gradual fill methods in order to render the animal insensible prior to their experience of pain. However, there is concern that the use of these gradual fill methods may increase the distress experienced by these animals. We evaluated social and anxiety behavior of rats that had been exposed to concentrations of CO_2_ that did not cause a loss of consciousness. We also evaluated the physiologic changes of rats that were euthanized with gradual fill protocols as compared to rapid fill methods. We found that rats exposed to concentrations of CO_2_ that did not cause a loss of consciousness did exhibit increased anxiety and decreased social behavior. We also found that the use of a 10% volume per minute displacement rate of CO_2_ resulted in physiologic and behavioral changes suggestive of distress.

**Abstract:**

Current recommendations for the use of CO_2_ as a euthanasia agent for rats require the use of gradual fill protocols (such as 10% to 30% volume displacement per minute) in order to render the animal insensible prior to exposure to levels of CO_2_ that are associated with pain. However, exposing rats to CO_2_, concentrations as low as 7% CO_2_ are reported to cause distress and 10%–20% CO_2_ induces panic-associated behavior and physiology, but loss of consciousness does not occur until CO_2_ concentrations are at least 40%. This suggests that the use of the currently recommended low flow volume per minute displacement rates create a situation where rats are exposed to concentrations of CO_2_ that induce anxiety, panic, and distress for prolonged periods of time. This study first characterized the response of male rats exposed to normoxic 20% CO_2_ for a prolonged period of time as compared to room air controls. It demonstrated that rats exposed to this experimental condition displayed clinical signs consistent with significantly increased panic-associated behavior and physiology during CO_2_ exposure. When atmospheric air was then again delivered, there was a robust increase in respiration rate that coincided with rats moving to the air intake. The rats exposed to CO_2_ also displayed behaviors consistent with increased anxiety in the behavioral testing that followed the exposure. Next, this study assessed the behavioral and physiologic responses of rats that were euthanized with 100% CO_2_ infused at 10%, 30%, or 100% volume per minute displacement rates. Analysis of the concentrations of CO_2_ and oxygen in the euthanasia chamber and the behavioral responses of the rats suggest that the use of the very low flow volume per minute displacement rate (10%) may prolong the duration of panicogenic ranges of ambient CO_2_, while the use of the higher flow volume per minute displacement rate (100%) increases agitation. Therefore, of the volume displacement per minute rates evaluated, this study suggests that 30% minimizes the potential pain and distress experienced by the animal.

## 1. Introduction

Euthanasia is defined as “the ending of the life of an individual animal in a way that minimizes or eliminates pain or distress” [1]. The use of animals as models in research frequently necessitates their euthanasia in order to collect samples and final data needed to support the studies. There is an ethical expectation that, if it has been determined that euthanasia is indicated, the method selected should be humane, taking into consideration the well-being of the animal that is being euthanized, in addition to the person who is performing the procedure [1]. Regulations in multiple countries require that the euthanasia of animals that are used in research be performed in a manner that minimizes pain and distress to the animal [2,3,4,5]. The American Veterinary Medical Association (AVMA) Panel on Euthanasia and Canadian Council on Animal Care in Science have published Guidelines that take into account existing literature and make recommendations on factors to be considered when determining the appropriate method of euthanasia for a particular species and circumstance [1,4]. 

Inhaled agents are frequently used to anesthetize animals prior to euthanasia. Indeed, it should be remembered that it is impossible to euthanize an animal using a chemical anesthetic agent (injected or inhaled) without first inducing anesthesia. Therefore, the current recommendations for the use of inhalant anesthetics should be considered when evaluating the use of inhalant gases for euthanasia. Tranquilli and Grimm provide an overview of the four stages of inhalant anesthesia. Stage I (the stage of voluntary movement) lasts from the initial administration of the inhalant anesthesia until the loss of the righting reflex. As they progress to Stage II, the animal becomes ataxic and loses its ability to stand. An animal in Stage II will exhibit involuntary movements and vocalizations, but it is no longer conscious. Stage III includes the level of anesthesia where surgery can be performed. In Stage IV, the animal has extreme central nervous system and cardiovascular depression and will die unless the anesthetic agent is removed and the animal is resuscitated [6]. When performing surgery, the veterinarian targets Stage III, while in euthanasia, they target Stage IV. As with the selection of an appropriate agent for euthanasia, the ideal anesthetic permits rapid induction, minimizing the potential pain and distress experienced by the animal as they progress through Stage I of anesthesia. 

Although the AVMA guidelines acknowledge that loss of consciousness (transition from stage I to stage II) will be more rapid if animals are initially exposed to a high concentration of an inhalant agent, they recommend gradual exposure because high concentrations of some gases, such as concentrations of CO_2_ at the levels of 40%–50%, can be aversive and distressing [1,7]. The AVMA Guidelines clearly state that high concentrations of CO_2_ are associated with pain, but suggest that the potential distress associated with exposure to low concentrations of CO_2_ over prolonged periods of time is not well characterized. Yet, the distressing effects of prolonged exposure to low concentrations of CO_2_ for rats are well documented [8]. Homeostatic regulation of blood CO_2_ concentrations is critical as CO_2_ alters blood pH by combining with water to form carbonic acid, and even a small change in blood pH can be fatal (for review, see [9,10]). Initial elevations of arterial CO_2_ (i.e., hypercapnia) lead to an increase in respiration rate and tidal volume to help “blow off” excess CO_2_ (see review [11]), but when arterial pH continues to decrease, a feeling of dyspnea and anxiety begin to emerge. This is followed by a feeling of fear and panic with moderate hypercapnia, and sedation and loss of consciousness during severe hypercapnia. For example, exposing humans to 5% CO_2_ increases respiration responses without increasing anxiety, while exposure to 7.5% CO_2_ increases anxiety symptoms (e.g., self-reports of fear and panic [12,13,14,15,16]). Exposure to 20% CO_2_ induces a strong sense of suffocation (dyspnea) that is accompanied by symptoms strongly associated with panic attacks (e.g., fear of dying, shortness of breath, cardiorespiratory response [17]). Anxiety symptoms, such as fear and panic can lead to aversion and avoidance (e.g., phobias), and although aversion/avoidance is not specific to anxiety-associated symptoms/emotions (e.g. aversion/avoidance can be related to food that can elicit disgust) [18]. In the presence of a life threatening event, panic can occur, but following the event fear and avoidance can occur. Fear is also associated with potential imminent threats [19]. 

Although it is impossible to assess dyspnea sensations and emotions in rodents in response to hypercapnia, panic-associated flight behaviors can be measured alongside cardiorespiratory responses that are associated with fear and panic responses in humans to give an indication of the aversive nature of a stimulus when it is presented (e.g., during CO_2_ exposure), and also an increase in fear and avoidance versus exploration-associated behaviors (i.e., conflict between a reward and a risk in a social interaction test or less time spent exploring open areas of an open field test) after the threat is gone, but still recent [20]. Similar to humans, exposing rats to mild hypercarbic gas (e.g., 7% to 7.5% CO_2_ [21]) increases respiration rate and tidal volume that reduces hypercapnia, but this concentration of CO_2_ does not alter aversion-associated exploratory behaviors in an open field test (unpublished observations). However, exposing rats to higher concentrations of hypercarbic gas (e.g., normoxic, 10% to 20% CO_2_) elicits additional components of a panic-associated response as evidenced by increases in sympathetic activity [22], blood pressure [20,23,24], anxiety-like behaviors as evidenced by increased aversion during risk versus reward conflict tests (i.e., Vogel test, open field exploration test, social interaction tests, and increases in panic/escape/flight-associated locomotor responses [20,23,25,26]), and mobilization of the hypothalamic-pituitary-adrenal (HPA) axis [27,28,29].

An additional and common misconception is that CO_2_ causes global central nervous system depression at low concentrations. Carbon dioxide readily crosses the blood-brain barrier to directly interact with neurons and inhibits the majority of neuronal networks causing a depression in activity which leads to loss of consciousness and anesthesia with rising concentrations. Yet, there are also specialized central chemoreceptive neurons [30,31] in medullary regions that are critical for regulating breathing following subtle changes in CO_2_/H^+^ [11] and in panic-generating brain regions, such as the hypothalamus [32]. In fact exposing healthy rats to normoxic 20% CO_2_ concentrations selectively increases cellular activity in panic and anxiety networks, such as the perifornical hypothalamus and dorsal periaqueductal gray [26]. When stimulated, these areas induce panic-associated symptoms in humans [33,34] and panic-associated behaviors and cardioexcitation in rats [35,36] and cats [37]. Others have proposed fear centers, such as the amygdala, as critical sites for CO_2_-induced panic in mice [38], but panic responses to 20% CO_2_ are intact in humans with bilateral loss of amygdala function [39]. 

In the first experiment, we exposed male rats to 5 min of premixed normoxic air containing 20% CO_2_, balanced with nitrogen, to characterize the development of panic-associated behaviors (social interaction and open field assessments) and physiology associated with these concentrations of CO_2_. This allowed evaluation of the potential distress experienced by rats that are exposed to the low concentrations of CO_2_ associated with gradual fill rates. In the second experiment, we assessed cardiovascular, locomotor, and core body temperature responses in a second group of male rats when euthanized with 100% CO_2_ infused at 10%, 30%, and 100% CO_2_ volume per minute displacement when there was no acclimation period prior to induction of anesthesia and subsequent euthanasia. This allowed evaluation of the behavior and physiology of rats being euthanized via current recommendations as compared to previous recommendations.

## 2. Materials and Methods

### 2.1. Experimental Overview

Experiment 1—This experiment was designed to measure the distress experienced by rats exposed to stable low concentrations of CO_2_. To accomplish this, rats were surgically implanted with radiotelemetry probes that are capable of measuring mean arterial blood pressure (MAP), heart rate, body temperature, and activity. Following recovery from surgery, baseline social interactions with an unfamiliar rat were assessed. Rats were then exposed to either atmospheric air or normoxic 20% CO_2_ for 5 min with telemetric recording of physiologic parameters before, during, and after exposure. Respiratory data was also collected using plethysmography. After the exposure, the rats were behaviorally assessed in an open field chamber and with a second social interaction with an unfamiliar rat. Details of these procedures are provided below. 

Experiment 2—This experiment was designed to evaluate the distress experienced by rats euthanized using 10%, 30%, and 100% volume per minute displacement rates of 100% CO_2_. For Experiment 2a, rats were surgically implanted with radiotelemetry probes that are capable of measuring MAP, heart rate, body temperature, and activity. Following recovery from surgery, each rat was placed into the induction chamber and 100% CO_2_ was infused at either a 10%, 30%, or 100% volume per minute displacement rate until death. During the euthanasia process, physiological data was collected using telemetry. Video data was also collected for behavioral assessment during the euthanasia process. For Experiment 2b, concentrations of CO_2_ and O_2_ were collected from the empty chamber using the same volume per minute displacement rates used in Experiment 2a. 

### 2.2. Methods

Description of measuring ambient CO_2_ and O_2_ concentrations during hypercarbic or atmospheric gas for in vitro Experiments 1 and 2b.

We previously used CO_2_ (ProCO_2_) and O_2_ (ProO_2_) sensors in our enclosed flow cages (30.5 cm width × 30.5 cm in. height × 61 cm in length) where we verified that O_2_ and CO_2_ concentrations remain normal with atmospheric air infusion, and that only the CO_2_ concentrations rapidly increase from <1% to 20% at the 5 min time point for CO_2_ challenge [40]. Here we used state of the art sensors to measure changes in the concentrations of CO_2_ (COZIR Ambientsensor, 0%–100% range with 3%+/− error, Gas Sensing Solutions, Cumbernauld, UK) and O_2_ (Luminox O_2_ UV light flux sensor, 0%–25% range with 2%+/− error, CO_2_ Meter, Inc., Osmond Beach, FL, USA) with the battery operated sensors within the cages interfaced with Gaslab data acquisition software (CO2 Meter, Inc., Osmond Beach, FL, USA) on a Windows operating system (Microsoft, Seattle, WA, USA). For Experiment 1, the measurements of ambient CO_2_ and O_2_ were collected during the animals’ exposure to the gases described for Experiment 1. For Experiment 2b, the measurements of ambient CO_2_ and O_2_ were collected from an empty chamber, but the gas was infused as described for Experiment 2a.

### 2.3. Animals and Housing Conditions for Experiments 1 and 2a

Adult male Sprague-Dawley rats (300–350 g, Harlan Laboratories, Indianapolis, IN, USA) were housed individually under standard environmental conditions (22 °C; 12/12 light/dark cycle; lights on at 7:00 AM) for 5–7 days prior to the surgical manipulations. Following surgical implantation of radiotelemetry probes (see next paragraph), all rats were individually housed to prevent the other rats from tampering with surgically implanted radiotelemetry. All rats were administered buprenorphine (0.05 mg/kg s.c.) every 12 h for a minimum of two days and were allowed to recover from surgery for at least five days prior to procedures. The rats were housed in shoebox caging with direct contact bedding (Sani-Chip, Harlan Industries, Indianapolis, IN, USA). Food (4% fat, 24% protein, 5% fiber, cat. no. 7001, Teklad, Madison WI, USA) and water were provided ad libitum. The colony was screened quarterly using indirect sentinels for the following pathogens: Kilham rat virus, *Mycoplasma pulmonis*, pneumonia virus of mice, rat coronavirus, reovirus-3, rat parvovirus, Sendai virus, transmissible murine encephalomyelitis virus, and ecto- and endo-parasites. The colony was free of all pathogens during this study. Animal care procedures were conducted in accordance with applicable federal regulations and following the review and approval by the IUPUI Institutional Animal Care and Use Committee (project 10623, approved 25 September 2013).

### 2.4. Respiratory Data Acquisition for Experiment 1 only

Digital signals from the pressure transducer were recorded with a Powerlab 8/30 data acquisition system (ADInstruments, Inc., Colorado Springs, CO, USA) and the digital signals were transformed and analyzed using LabChart Pro software (ADInstruments, Inc., Colorado Springs, CO, USA). 

### 2.5. Surgical Procedures for Radiotelemetry Probe Implantation and Data Acquisition for Experiments 1 and 2a

Prior to surgery, rats were anesthetized with 2 to 5% isoflurane (Piramal Critical Care, Inc., Bethlehem, PA, USA) with a nose cone connected to a precision vaporizer (MGX Research Machine; Vetamic, Rossville, IN, USA) during the surgery. All rats were fitted with femoral arterial catheters for measurement of mean arterial blood pressure (MAP) and heart rate (HR) as previously described [41]. This catheter was connected to a telemetric probe that was aseptically implanted into the peritoneal cavity. The telemetry probe also contained an internal thermistor for measuring core body temperature and is also capable of assessing general locomotor activity (Cat. no. HD-S11, Data Sciences International (DSI), St. Paul, MN, USA). The temperature sensor of this probe has an accuracy of 0.1 °C; a resolution of 0.05 °C; and an operational range of 34 to 41 °C. 

### 2.6. Experimental Procedures for Gas Infusions for Experiments 1 and 2a

Experiment 1—We have previously shown that challenging rats with 5 min infusions of normoxic 20% CO_2_ gas consistently induces equivalent cardiovascular and anxiety behavioral responses when there is at least 48 h between challenges [20]. Thus, in a counter-balanced design all rats received one infusion of either atmospheric air or normoxic 20% CO_2_ with at least 48 h between treatments. Rats were placed in a clear custom-built Plexiglass^®^ cylindrical plethysmograph chamber where they were capable of moving (i.d. 9.5 cm, length 26 cm, wall thickness 3 mm) with atmospheric air infused at a flow rate of 2.8 L/min using a Vetamac flowmeter (Rossville, IN, USA) until a steady baseline respiration rate was noted (approximately 30 min). A plastic T-connector was inserted 20 cm away from the start of the output line and then linked to one input of a differential pressure amplifier (model 24PC01SMT, Honeywell Sensing and Control, Golden Valley, MN, USA), the second input being opened to the room air. The atmospheric infusion rate was sufficient to prevent any rise of CO_2_ in the plethysmograph [20]. Radiotelemetry probes were turned on with a magnet placed near abdomen, and receivers were placed below the experimental gas infusion cages in order to acquire radiotelemetry data during procedures. All rats had infusions of the following: (1) 5 min infusion of atmospheric gas (<1% CO_2_, 21% O_2_, 78% N_2_: Praxair, Inc., Indianapolis, IN, USA) for baseline measurements; then (2) either the control gas or experimental normoxic, hypercarbic gas (20% CO_2_, 21% O_2_, 59% N_2_: Praxair, Inc., Indianapolis, IN, USA) for 5 min, and finally; (3) 5 min infusion of atmospheric gas. 

Experiment 2a—In a separate group of rats than experiment 1, individual rats were placed into enclosed flow cages (30.5 cm width × 30.5 cm in. height × 61 cm in. length) for 5 min, then the treatment group of gas infusions of atmospheric air (<1% CO_2_, 21% O_2_, 79% N_2_: Praxair, Inc., Indianapolis, IN, USA) or one of 3 different CO_2_ volume per minute displacement methods where 100% CO_2_ was infused at 10%, 30%, or 100% volume per minute displacement (100% CO_2_, Praxair, Inc., Indianapolis, IN, USA), was initiated to euthanize the animals. 

### 2.7. Behavioral Testing Post-Gas Infusions Only for Experiment 1

Following exposure to hypercarbic and atmospheric air gases, rats were immediately placed in the open-field box for a 5 min period, then assessed in a social interaction test for 5 min. The open-field arena was 90 cm × 90 cm, with 40 cm walls that was divided into a 6 × 6 grid of equally-sized squares with four squares forming the center. The test started by placing a rat in the center, and the following parameters were scored (distance traveled, time and frequency of time spent in center grid (four squares), and middle regions (12 squares surrounding center grid)). The social interaction (SI) test was done in the open field arena and is a fully validated test of experimental anxiety-like behavior in rats [20,36]. The “experimental” rat and an unfamiliar “partner” rat are both placed in the center of the box, and the total duration (s) of non-aggressive physical contact (grooming, sniffing, crawling over and under the partner rat) initiated by the “experimental” rat is quantified over 5 min. Baseline social interaction was done with an unfamiliar partner rat and on the test day this was repeated with a different unfamiliar partner rat. All behaviors were videotaped and the sessions were scored using ANY-maze for open field (Stoelting, Wood Dale, IL, USA), or by Stephanie Fitz (who was blind to treatments) for social interaction.

### 2.8. Behavioral Testing just Prior to and during CO_2_ Euthanasia Gas Infusion Only for Experiment 2a

Video data was collected using a video camera (cat. no. DMK23U618, Imaging Source, Charlotte, NC, USA) that was connected to a PC for recording. The recordings were scored offline by Cristian Bernabe for time duration of seizure (rapid and repeated contraction and relaxation of body muscles with uncontrolled shaking of the body), running (rapid movement within the cage), and gasping (sudden and sharp inhalation of air through the mouth) behaviors using ODLog™ software (version 2.5 Macropod Software, Yarraville, Victoria, Australia). Additionally, the video recordings were used to approximate the time to loss of consciousness. In humans, loss of consciousness is associated with the inability to engage in verbal communication [1]. In animals, the loss of righting reflex is utilized [6]. This can correlate to the ataxia seen in stage I of anesthesia, but as involuntary movement can continue in stage II, where the animal is unconscious, care must be taken in evaluation of movement as an indicator of consciousness [6]. For this study, we determined that the rats had lost consciousness at the point when gross motor control was compromised. We had initially determined that the point when the rat let its nose touch the cage bottom would be the time for loss of consciousness, but this behavior was inconsistent in the groups. So, instead we considered the gross loss of motor function as the point where the rat either touched its nose to the ground or began staggering with significant ataxia. It is recognized that both of these measures are very subjective and may not be reflective of the actual state of consciousness of the animals. The video was recorded at baseline and from the initiation of the infusion of gas and was observed for 5 min or until the respiration ceased. 

### 2.9. Statistical Analyses for Experiments 1, 2a, and 2b

Dependent variables for analyses of CO_2_ and O_2_ (experiments 1 & 2b) concentrations and in vivo cardiovascular (HR, MAP), respiratory, locomotor activity, and core body temperature (Experiments 1 and 2a) were analyzed using an ANOVA with gas as the main factor and for gas concentrations and physiological parameters with time as the repeated measure. An ANOVA was utilized to analyze differences between groups for social interaction behaviors, and a two-tailed paired *t*-test was used to analyze open field behaviors. A Levene’s Test of Equality of Error Variance was also done to determine equal variances in the groups. In the presence of significant main effects or main effect × time interactions of ANOVAs, we initially assessed between subjects effects using a Fisher’s Least Significant Difference (LSD), protected by ANOVAs at each time point (when relevant), was used for post hoc analyses. For over time data, we then assessed within subjects over time effects with a Dunnett’s post hoc test with t−1 as the baseline. The alpha level was set at 0.05. All statistical analyses were carried out using Graphpad Prizm 6 (Graphpad, Inc., La Jolla, CA, USA) and SPSS 14.0 (SPSS Inc., Chicago, IL, USA), and all graphs were generated using SigmaPlot 2001 (SPSS Inc., Chicago, IL, USA) or Graphpad Prism 6 (Graphpad, Inc., La Jolla, CA, USA), and an illustration program (CorelDraw X5 for Windows, Viglen Ltd., Alperton, UK).

## 3. Results

**Figure 1:**
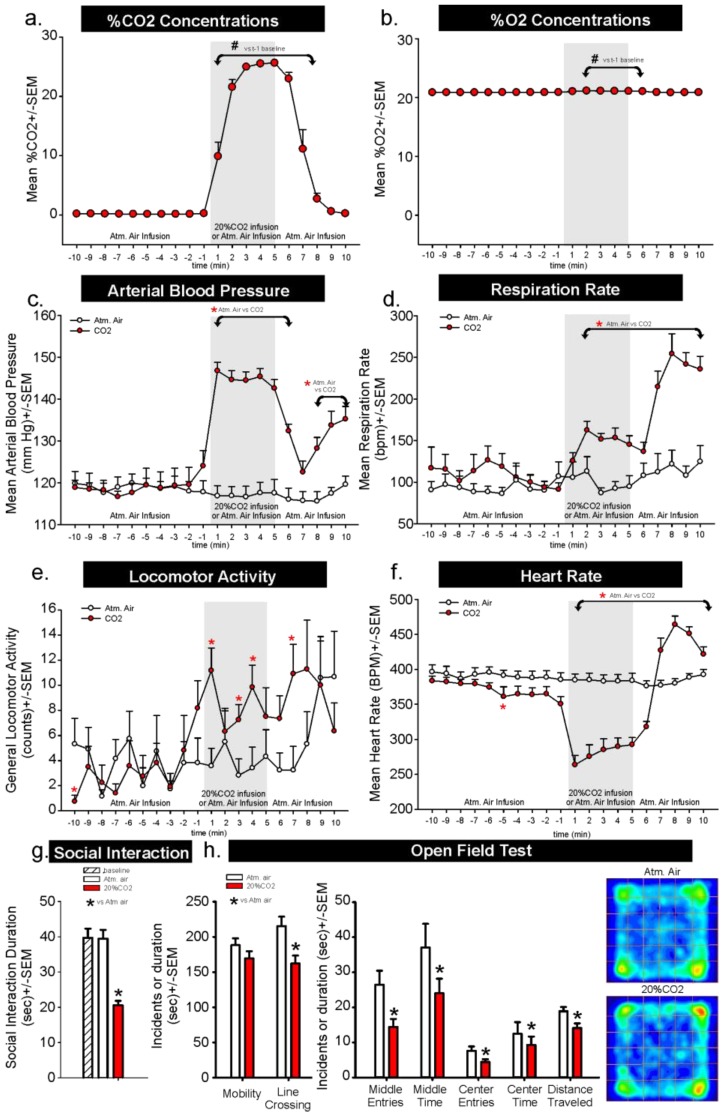
Line graphs in (**a**–**f**) respectively represent ambient concentrations of CO_2_ and O_2_; mean arterial blood pressure (mmHg); respiration rate (breaths per minute); general locomotor activity; and heart rate (beats per minute) at baseline during 10 min atm. air infusion (all rats), then also during either a 5 min atm. air challenge or 20% CO_2_ challenge (gray shaded region) following by all rats receiving another 5 min atm. * represents between-subject significance *p* < 0.05 with a Fisher’s LSD post hoc test protected by a significant one way ANOVA with gas treatment as the main factor and time as a repeated measure. ^#^ symbol in a-b represent significant *p* <0 .05 within subject over time effects (against t-1 min) from a Dunnett’s post hoc test. * symbol in (**e**) represents between subjects significance *p* < 0.05 with a two-tailed Dunnett’s post hoc test protected by a significant one-way ANOVA with gas treatment as the main factor. * in (**f**) represents between-subject significance with a two-tailed paired *t*-test. Behavior testing immediately following gas challenges were (**g**) social interaction duration, and (**h**) open field behaviors.

In Experiment 1, for assessments of CO_2_ concentration changes within the chamber following a 5 min infusion of normoxic 20% CO_2_ we observed a significant over time effect (F_(19,114)_ = 105.2, *p* < 0.001, Figure 1a, *n* = 7). In this analysis there was a rapid increase in CO_2_ concentrations from undetectable to 10% within 1 min, and 20% to 24% at 2–6 min post-infusion, which returned to 3% to 0% 3–5 min post-infusion of atmospheric air. It is noteworthy that the infusions of a custom mixed normoxic 20% CO_2_ tank led to approximately 24% CO_2_ concentrations which could be due to the range error of the sensor, which was +/−3% and/or errors in tank preparations. For assessments of O_2_ concentration changes within the chamber following a 5 min infusion of normoxic 20% CO_2_, we also observed a significant over time effect (F_(19,114)_ = 35.2, *p* < 0.001, Figure 1b, *n* = 7). In this analysis O_2_ concentrations increased from 20.9% to 21.1% within 1 min, then returned to 20.9% 7 min post-infusion which could also be due to the range error of sensor, which is +/−2% and/or errors in tank preparations. 

Compared to atmospheric air infusions, infusing normoxic, 20% CO_2_ into a small volume chamber induced a robust and immediate pressor response in the rats that went from baseline means of approximately 120 mmHg to 145 mmHg (*gas × time interaction* F_(19,399)_ = 42.1, *p* < 0.001, Figure 1c, *n* = 12,11). The pressor response began to return to normal post-CO_2_ infusion, but then increased again. This coincided with a dramatic increase in respiration rate in Figure 1d when we noted that the rats had increased respiration rates during CO_2_ infusion but then, at offset, were moving to the atmospheric air intake, where they began to breathe rapidly (*gas × time interaction* F_(19,361)_ = 24.5, *p* < 0.001, Figure 1d, *n* = 11,10, we lost signal on one rat in each group). Locomotor activity increased post CO_2_ infusion and remained increased even at offset (*gas × time interaction* F_(19,399)_ = 2.3, *p* = 0.002, Figure 1e, *n* = 12,11). Dramatic reflexive bradycardia responses post-CO_2_ returned to baseline at offset of CO_2_, and became tachycardic shortly after (*gas × time interaction* F_(19,399)_ = 53.7, *p* < 0.001, Figure 1f, *n* = 12,11). Behaviors assessed immediately following air challenges revealed that CO_2_-challenged rats had reduced social interaction durations, compared to atmospheric air challenged rats (F_(2,33)_ = 24.4, *p* < 0.001, Figure 1g, *n* = 12,12,12), and increased thigmotaxis in the open field test (line crossing t_(10)_ = 4.1, *p* = 0.002, middle entries t_(10)_ = −12.1, *p* < 0.001, middle time t_(10)_ = −10.1, *p* < 0.001, center entries t_(10)_ = −13.8, *p* < 0.001, center time t_(10)_ = −13.0, *p* < 0.001, distance traveled t_(10)_ = −13.1, *p* < 0.001, Figure 1h, *n* = 11,11, video malfunction on one rat, other crossover removed for paired *t*-test analyses). 

**Figure 2:**
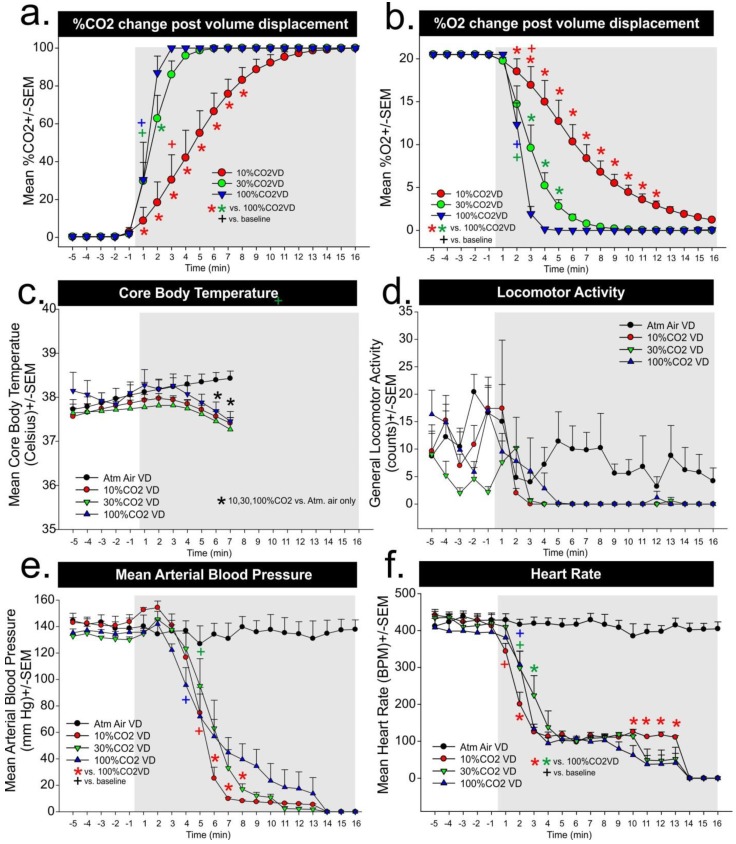
Line graphs in (**a**–**f**) respectively represent ambient concentrations of CO_2_ and O_2_; core body temperature (Celsius); general locomotor activity (counts/min); mean arterial blood pressure (mmHg); and heart rate (beats per minute) when placed in volume displacement cage for 5 min prior to either infusions of atmospheric air (Atm air), or 10%, 30%, or 100% CO_2_ volume displacement per minute procedures (gray shaded region). * represents between-subject significance *p* < 0.05 with a Fisher’s LSD post hoc test protected by a significant one-way ANOVA with gas treatment as the main factor and time as a repeated measure.

In Experiment 2b, for assessments of CO_2_ concentration changes within the chamber following 10, 30% and 100% CO_2_ volume per minute displacement rates, we observed a significant gas infusion × time interaction (F_(40,180)_ = 8.0, *p* < 0.001, Figure 2a, *n* = 4,4). For the 100% CO_2_ volume per minute displacement rate there was a rapid increase in CO_2_ concentrations from undetectable to 30% and 90% 1–2 min post-infusion, respectively. For 30% volume displacement this increase only significantly lagged by 1 min, but with 10% CO_2_ volume displacement it took 4 min for CO_2_ concentrations to be >40%, and the chamber reached 100% CO_2_ 11 to 12 min post-infusions. For in vitro assessments of O_2_ concentration changes following 10%, 30% and 100% CO_2_ volume per minute displacement we observed a significant gas infusion × time interaction (F_(40,180)_ = 12.4, *p* < 0.001, Figure 2b, *n* = 4,4). In this analysis, with 100% CO_2_ volume displacement there was a rapid decrease in O_2_ concentrations from approximately 21% O_2_ to 12.5% and 2% O_2_ within 1–2 min, respectively. For 30% volume per minute displacement, the decrease was slower and it took 5 min to reach approximately 2% O_2_. With 10% CO_2_ volume per minute displacement, it took 5 min for CO_2_ concentrations to be 12.5%, and the chamber reached 2% O_2_ at 13–14 min post-infusion.

In Experiment 2a, atmospheric air response was included in Figure 1 figures c–f, but was not included in analyses. For the core body temperature there was a significant gas × time interaction (F_(36,204)_ = 6.8, *p* < 0.0001, Figure 2c, *n* = 5,5,5,6) with core body temperature beginning to decrease at 5 min post 10%, 30%, and 100% CO_2_ volume per minute replacement, respectively, and significant differences as compared to atmospheric air evident at 6 and 7 min. For locomotor activity, there was no gas × time interaction (F_(40,260)_ = 1.3, *p* = 0.103, Figure 2d, *n* = 5,5,5,6), and although an significant time effect was noted (F_(20,260)_ = 10.4, *p* < 0.0001), post hoc analyses failed to detect changes from baseline. This could be due to the agitated motor activity at baseline between groups which ranged from 2–10 counts/min compared to a range of 2–6 counts/min in the baseline of Figure 1e. For MAP there was a significant difference in the gas × time interaction (F_(40,260)_ = 1.6, *p* = 0.016, Figure 2e, *n* = 5,5,5,6). It is worth noting that baseline MAP is 130 to 145 mmHg, which is 10 to 25 mmHg above baselines seen in Figure 1a, and similar to MAP see post-normoxic 20% CO_2_ exposure. Assessment of over time within subjects’ effects on MAP revealed there was a significant decrease in MAP 4 min post 100% CO_2_ volume displacement and this took a minute longer for the 10% and 30% CO_2_ volume per minute displacement methods. The MAP for rats in the 10% CO_2_ volume per minute displacement group was significantly decreased as compared to the other groups beginning around the sixth minute. For heart rate, there was a significant gas × time interaction (F_(40,260)_ = 1.7, *p* = 0.004, Figure 2f, *n* = 5,5,5,6). Assessment of over time effects revealed that 10%, 30%, and 100% CO_2_ volume per minute displacement led to a rapid reflexive bradycardia within 1–2 min in all groups (see + symbol in Figure 2f). There were between subjects differences noted, but they were primarily occurring 10–14 min post-gas infusions. 

**Figure 3:**
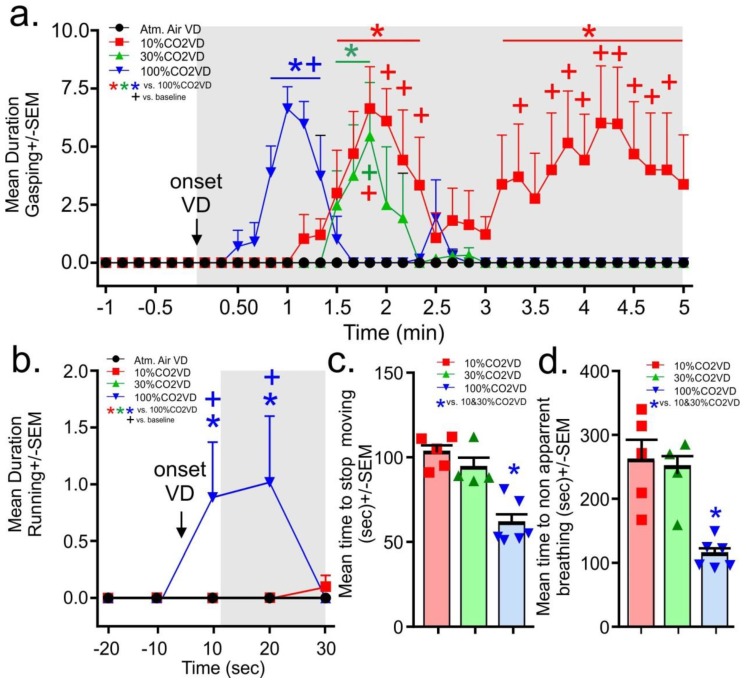
Line graphs in (**a,b**), respectively, represent the mean duration of gasping behaviors and running behaviors just prior to and following gas VP (gray shaded region). * represents between-subject significance *p* < 0.05 with a Fisher’s LSD post hoc test protected by a significant one-way ANOVA with gas treatment as the main factor and time as a repeated measure; (**c**) bar graph represents the mean time before the rat stopped moving; and (**d**) bar graph represents the mean time before breathing became non apparent. Individual points in c-d represent actual values of each animal. * represents a Fisher’s LSD post hoc test protected by a significant ANOVA.

From the evaluation of the video recordings, the gasping behaviors did not occur prior to gas infusions. Upon initiation of the infusion of the 100% CO_2_, the rats in the 100% volume displacement per minute group displayed significant gasping in less than a minute. This gasping was significantly increased for 30 s (gas × time interaction F_(105,595)_ = 3.7, *p* < 0.0001, Figure 3a, *n* = 5,5,4,6 one video malfunctioned for 30% group). In the 10% and 30% volume displacement per minute treatment groups, the onset of gasping occurred at approximately 1.5 min post-gas initiation, with the 30% volume displacement per minute group gasping for about 20 s. The 10% volume displacement per minute group gasped for 1 min, then stopped for approximately 1 min before resuming and persisting until the mean time for breathing to stop being apparent for all rats. Only the 100% volume displacement per minute group displayed running behaviors and these occurred only in the first 20 s post-gas infusions (gas × time interaction F_(12,64)_ = 2.3, *p* = 0.016, Figure 3b *n* = 5,5,4,6). No seizures were noted in any animals.

For the determination of approximate loss of consciousness, the time to stagger or touch the nose was combined for all rats as not all rats consistently engaged in these behaviors. It took approximately 60–80 s for rats euthanized with the 10% and 30% volume per minute displacement rate to display the loss of gross motor coordination associated with staggering and dropping the head, Rats euthanized with the 100% volume per minute displacement rate displayed this loss of gross motor coordination by 30 s (the 100%CO2VD was only significantly lower than the 30%CO2VD group, data not shown, F(2,12) = 4.3, *p* = 0.040, *n* = 5,4,6). Rats euthanized by the 10% and 30% volume per minute displacement rates stopped moving at approximately 100 s, while rats euthanized with the 100% volume per minute displacement rate stopped moving at approximately 60 s (Figure 3c, *n* = 5,4,6 F(2,12) = 19.3, *p* = 0.0002). Breathing became non apparent first for the 100% volume displacement per minute treatment group at approximately 2 min and at 4 min for the other volume displacement per minute treatment groups (F(2,12) = 12.7, *p* = 0.001, Figure 3d *n* = 5,4,6). 

## 4. Discussion

In the first experiment, we first challenged healthy rats to a 5 min infusion of normoxic 20% CO_2_ to isolate behavioral and physiological responses to increased CO_2_ concentration. This method only increased ambient O_2_ concentrations by approximately 0.2%, but rapidly increased ambient CO_2_ concentrations from being undetectable to 20% to 24% during the infusions. Behavioral assessments revealed that rats experiencing the 20% CO_2_ challenge had increased panic and escape or flight associated locomotor responses (from 2 counts/min to 12 counts/min) during the CO_2_ challenge. This challenge also increased mean arterial blood pressure by 25 to 28 mmHg, and respiration rate by approximately 75 breath/min. At the offset of CO_2_ infusions and onset of atmospheric air infusions, we noted that the rats would orient themselves so that their nose was near the gas infusion intake which coincided with an even greater increase in respiration from 150 breaths/min to almost 275 breaths/min. Following the CO_2_ challenge, the rats expressed increased fear and avoidance-associated behavior (operantly defined as thigmotaxis and social avoidance, and evidenced by reduced time spent exploring center regions of an open field arena and reduced social interaction time). It is possible that CO_2_ induced drowsiness that might have contributed to a decrease in the exploratory behavior in these rats, but previous work with rats that were fully anesthetized with CO_2_ report that the rats regain locomotor control within 90 s and exhibit a complete clinical recovery from anesthesia within 5 min, but they exhibit antinociception for up to 60 min [42]. Environmental concentrations of CO_2_ that are less than 5% do not typically induce symptoms and signs of anxiety in people [12], though there is an increase in the respiratory activity to attempt to blow off excessive CO_2_ to return the plasma CO_2_/H^+^ to homeostasis (for review, see [9,10]). However, environmental concentrations of 7.5% CO_2_ begin to signal air hunger and are anxiety symptom/sign provoking in humans [12], and environmental concentrations of 20% CO_2_ induce symptoms such as suffocation sensations, fear of dying, and robust cardio-respiratory responses which are all strongly associated with panic attacks [17]. These human findings are fairly consistent with a study where gradually filling a test chamber up to 10% CO_2_ did not significantly alter rat behavior, but when concentrations approached 15% there was a reduced latency to leave the test chamber, and at 20% the majority of rats left immediately [43]. The relationship between CO_2_ and panic attacks is further supported in that exposing patients with panic disorder to 5%–7% CO_2_ will provoke panic attacks in the majority of these patients and no panic attacks in healthy controls [44,45,46]. Overall this provides further evidence of the aversive nature of exposure to 7.5%–20% CO_2_. 

In the second experiment, we sought to measure CO_2_ and O_2_ concentrations during either atmospheric air infusions of a 10%, 30%, and 100% CO_2_ volume per minute displacement euthanasia protocol while measuring locomotor behavior, and physiological parameters. In the evaluation of chamber gas concentrations for Experiment 2b, both the 30% and 100% CO_2_ volume per minute displacement methods led to rapid increases in ambient CO_2_ concentrations, which increased to 25% within 1 min. Both reached greater than 90% within 4 min and there were minimal differences in the rate of change. However the 10% CO_2_ volume per minute displacement method led to a gradual increase in ambient CO_2_ concentration that was approximately 7%–20% within the first 2 min and 30% within 3 min, which suggests these rats remained in panic inducing ranges of CO_2_ concentrations significantly longer than the other higher CO_2_ displacement methods. All of the methods led to fairly rapid decreases in blood pressure within 4 min for 100%, and 5 min for 10% and 30%, and a decrease in heart rate within 1 min for 10% and 2 min for 30% and 100%.

The core body temperature readings may need to be interpreted with caution. The telemetry probes have an operational range of 34 to 41 °C, so any temperatures that are below 34 °C are no longer accurate. However, we have a manuscript in resubmission clearly demonstrating that the 5 min 20% CO_2_ exposure used here elicits a robust 7–9 °C increase in tail temperatures (due to active vasodilation of tail artery) that precedes the decrease in core temperature. Thus, this drop in core body temperature is not just due to a cessation of metabolic and locomotor activity, but an active cooling mechanism. This hypothesis is consistent with human and rodent data showing that CO_2_ has local cutaneous vasodilatory action [47,48]. 

Our evaluations of CO_2_ concentrations are consistent with findings that were previously reported regarding how the concentration of CO_2_ changes in the chamber over time with constant volume per minute displacement flow rates [1,49]. Of concern, when using a volume per minute displacement rate of 10%, the environmental concentrations of CO_2_ reached levels associated with anxiety within 1 min of the initiation of the CO_2_ flow. The concentrations of 40%–50%, which have been demonstrated to induce loss of consciousness [1,7], were not achieved until approximately 5 min after the initiation of the CO_2_ flow. This meant that the animal would be exposed to anxiety- and panic-inducing concentrations of CO_2_, without loss of consciousness, for at least 4 min. Contrast this with the volume per minute displacement rate of 30%. In this case, the anxiety inducing threshold was also achieved within 1 min of the initiation of CO_2_ flow, but the environmental concentration of CO_2_ exceeded 50% approximately 2 min after the initiation of CO_2_ flow. In this case, the animal would be exposed to anxiety- and panic-inducing concentrations of CO_2_, without loss of consciousness, for approximately 1 min. This interval was shortened further when a 100% volume per minute displacement flow rate was utilized. However, there is evidence that this could increase the opportunities for exposure to painful concentrations of CO_2_, where carbonic acid has increased high enough in the respiratory tract to interact with nociceptors to signal a burning sensation [8]. For example, humans inhaling air with 50%–60% CO_2_ were most likely to call the experience unpleasant [50], but inhalations of 80%–100% CO_2_ were more often reported as painful with self-reports of a burning sensation and difficulty inhaling the gas [8]. Within the same manuscript, the authors exposed rats to different concentrations of CO_2_ (60%, 70%, 80% and 100%) until anesthesia or euthanization occurred. They reported that the most aversive behaviors (seizures) occurred in lower concentrations of CO_2_ exposure and were absent to mild with 100% CO_2_ in most instances. They further noted that trauma to airways and nasal cavities occurred in these lower concentrations of CO_2_, as well, though it is not clear if the animals were conscious when the trauma occurred. Thus, the short exposure humans had to CO_2_ may have led to a fast aversive response [51], but prolonged exposure (anesthesia inducing) to lower concentrations of CO_2_ could produce enough carbonic acid to elicit similar burning and pain as high concentrations of CO_2_ that would likely be prolonged since time to anesthesia would be delayed, but this has not been explored. In all of these cases, it is necessary to balance the potential for pain (likely higher for a short period of time with a 100% volume per minute displacement rate) with the possibility for distress (likely higher for a prolonged period of time with a 10% volume per minute displacement rate). The lower the flow of the volume per minute displacement rate, the more time the animal would be left in concentrations of gas that are well-documented to cause anxiety and panic prior to loss of consciousness, which occurs at concentrations of 40%–50%, dependent upon equilibration of the CO_2_ gas [1,7]. This strongly suggests that while the low flow volume per minute displacement rates may minimize pain, they increase the distress experienced by the animal.

Evaluation of the behavioral assessment of loss of consciousness in this study provided a less clear picture. With the available tools, defining the exact time of loss of consciousness is challenging because we lack the ability to create a clear determination of when this loss of consciousness has occurred. In humans, loss of consciousness is generally accepted to have occurred at the time when the individual is no longer able to engage in verbal communication [6], but this definition is compounded by the fact that humans can engage in significant and appropriate verbal communication with others that is not recalled when under the effect of alcohol or drugs [52,53]. It has been suggested that electroencephalography (EEG) can be used to assess consciousness in animals [1,54,55], but as EEG measurements are present in anesthetized and sleeping animals, this assessment tool is not necessarily as specific as desired [54,55,56]. In animals, the loss of righting reflex is generally accepted as the marker of loss of consciousness [1,6], but again, pinpointing this exact moment is difficult, especially when using gaseous agents where opening the chamber to test the animal affects the concentration and distribution of the gas. If the ability to handle the animal is built into the cage (as was done in one study [57]), this could lead to more reliable assessments of the loss of righting reflex. 

For this study, we determined that the rats had loss of consciousness at the point when gross motor control was compromised. We had initially determined that the point when the rat let its nose touch the cage bottom would be the time for loss of consciousness, but this behavior was inconsistent in the groups. Instead we considered the gross loss of motor function as the point where the rat either touched its nose to the ground or began staggering with significant ataxia. It is recognized that both of these measures are very subjective and may not be reflective of the actual state of consciousness of the animals. However, it is worth noting that the interval to achieve this gross loss of motor control was significantly less than anticipated based on the measurements in the empty chamber. For example, in the empty chamber, we projected that the rat would be conscious for as long as 4–5 min when euthanized using a 10% volume pre minute displacement rate, but our behavioral data suggested that the loss of consciousness occurred closer to 1.5–2 min, though the behavioral data of the rat euthanized with a 100% volume per minute displacement rate was consistent with the predicted loss of consciousness around 25 s. Aside from the concern about the subjective definition of loss of consciousness affecting this discrepancy, it should also be noted that the presence of the animal in the chamber would create turbulence because of its movements and thermal load which could lead to better mixing of the carbon dioxide to facilitate exposure that would not be seen in the empty chamber.

Environmental concentrations of oxygen may also have an effect on the distress experienced by an animal. For humans, during suffocation, CO_2_ increases and O_2_ decreases, stimulating an air hunger response with compensatory respiratory and cardiovascular responses [46]. When the levels of CO_2_ are increased to 20%, but oxygen levels are kept stable at 21%, an air hunger panic is evidenced by a strong sense of suffocation (dyspnea) that is accompanied by symptoms strongly associated with panic attacks (e.g., fear of dying, shortness of breath, and cardioexcitation [17,58]). Here, in rats, we also see increased anxiety-associated behavior and escape- or flight-associated behaviors that coincide with robust cardiorespiratory responses following exposure to 20% CO_2_ in normoxic air. Thus, being exposed to high concentrations of CO_2_ in the absence of any change in O_2_ is clearly panicogenic. 

In the second experiment we utilized volume displacement methods that increased CO_2_ but also subsequently decreased O_2_ levels and the rats appeared to be less distressed than in Experiment 1. Adding oxygen to the euthanasia chamber when using carbon dioxide may not minimize distress, but may actually increase the distress experienced by the animal [1]. As demonstrated by the oxygen measurements in the empty euthanasia chamber during the infusion of CO_2_ gas, the 10% volume per minute displacement rate results in a very slow loss of oxygen. Therefore, in addition to the animals being exposed to concentrations of CO_2_ that are anxiety inducing, their cardiovascular and respiratory functions are kept in a manner that induces an acute air hunger, as well. In contrast, when the 30% and 100% volume per minute displacement flow rates for CO_2_ are utilized, there is an acute drop off of oxygen over the first 2–3 min. This decrease in oxygen would be expected to induce a “drowsiness” response in the animals, further decreasing the potential for distress associated with these volume per minute displacement rates of CO_2_. Currently, this is only an observation, and a future study comparing CO_2_ displacement concentrations in normoxic, hypoxic, and hyperoxic conditions would be needed to test this hypothesis.

Examination of the live animal data collected in the second study demonstrates that the events leading up to the euthanasia process itself are stressful events for the animal. A typical calm rat will have a mean arterial pressure of approximately 120 mmHg and a heart rate of 360 to 380 beats per minute. Historically, our laboratory has seen mean arterial blood pressures of 140 mmHg and heart rates of 400 to 450 beats per minute during a panic episode induced by exposure to 20% CO_2_ [20,23]. In this data, the animals were all exhibiting these elevated levels during the 5 min that they were in the chamber prior to induction of the euthanasia process. This is likely explained because, in this study, rats were removed from their home cage and placed into the novel euthanasia chamber. Although it is generally recommended that animals remain in their home cage when being euthanized, it should be recognized that this practice is variable across institutions and actively discouraged during the induction of anesthesia with other inhalant gases (such as isoflurane) for survival procedures. It should also be recognized that there are often other stressors, such as the stress of transport or comingling of groups of animals, that are also frequently done at the time of euthanasia. So, although current best practices for euthanasia suggest that it is preferential to transport the home cage containing the animal to the euthanasia chamber [59] and let the animal acclimate to its new surroundings for at least 30 min prior to initiating the euthanasia procedures (as demonstrated by the first study presented here), this is not uniformly recognized. 

Since the events leading up to the actual euthanasia of the rats were already inducing a marked stress response, it is difficult to distinguish significant differences in the cardiovascular response to the varying volume per minute displacement rates of CO_2_. However, there was an increase in the mean arterial pressure of the animals that were euthanized using the 10% volume per minute displacement rate in the first 2 min following the initiation of the CO_2_ gas infusion. This is suggestive of increased sympathetic activity and distress experienced by the animal. At 3 min post-induction of CO_2_, the mean arterial pressure of the rats exposed to the 100% volume per minute displacement rate of CO_2_ drops below baseline, but this drop does not occur until approximately 4 min for the other two volume per minute displacement rate groups. This is likely due to the delayed onset of freezing behaviors that would be expected in the rats exposed to the lower volume per minute displacement flow rates, though these behaviors were not seen in the rats euthanized for this study. 

Locomotion of the euthanized animals following the introduction of CO_2_ is difficult to interpret since the placement of the non-handled rats into a novel chamber appeared to produce active, but variable, locomotor responses. Although there were no significant changes in pattern between the treatment groups, the 30% and 100% CO_2_ and the 10% CO_2_ groups, respectively, displayed prolonged inactivity within 3 and 5 min post displacement. The significance of this drop in locomotion could be representative of a loss of consciousness, though we did not attempt to characterize loss of consciousness in this study. Loss of consciousness for humans is defined as the point where the person can no longer verbally respond, but for animals, the loss of righting reflex is generally accepted as the point of loss of consciousness [1]. However, it is challenging to determine exactly when this point occurs because different observers may determine that the animal has lost its ability to right itself at different times. 

Subsequent assessments of behavior were revealing. The rats that were euthanized using the 10% volume displacement per minute rate displayed a prolonged period of gasping behavior that began around 1.5 min (when the concentration of CO_2_ in the chamber would have exceeded 10%) and extended beyond 4 min (when the concentration of CO_2_ in the chamber would have exceeded 40%). As other studies have demonstrated that concentrations of at least 40% CO_2_ are required to induce a loss of consciousness (dependent upon chamber conditions and definition of loss of consciousness used by the study), these animals were clearly demonstrating dyspnea and distress during this period of time. The gasping behaviors demonstrated by the 30% and 100% volume displacement per minute treatment groups were similar and significantly shorter in duration.

Only the rats exposed to the 100% volume displacement per minute showed a marked increase in running at onset of the gas which is suggestive of immediate panic and likely speaks to attempts to escape from the aversive nature of the gas (e.g., dyspnea and burning sensations). This behavior further supports that high volume displacement rates do not minimize pain or distress in the euthanasia of rats.

## 5. Conclusions

Carbon dioxide anesthesia overdose is an important and appropriate method of euthanasia for laboratory rats, but refinements are necessary to ensure that the pain and distress experienced by the rat is minimized. The use of volume per minute displacement flow rates instead of precharged chambers when using carbon dioxide for euthanasia is critical to prevent the exposure of animals to noxious stimuli that can result in pain. However, the results of this study suggest that the use of very low volume per minute displacement flow rates (such as 10% volume displacement per minute) for carbon dioxide in rats has the potential to significantly increase the distress experienced by the animals. This distress is induced by the prolonged period of time that the rats are exposed to concentrations of CO_2_ that are known to induce panic and anxiety and the concurrent stabilization of oxygen levels in the chamber, which is known to induce air hunger in the face of elevations of CO_2_. The elevations in mean arterial blood pressure of the rats euthanized with the 10% volume per minute displacement rate beyond the elevations that were already induced by the stress of transport and novel environment support this finding. Alternatively, the use of medium to high volume per minute displacement flow rates result in a minimizing of potential distress experienced by the rat through the shortening of the period of time that the rats are exposed to concentrations of CO_2_ that are known to induce panic and anxiety and the rapid depletion of O_2_, which is known to induce a drowsiness prior to loss of consciousness. Therefore, the results of this study suggest that volume per minute displacement rates of 10% should not be used as there is increased potential exposure to pain and distress associated with prolonged exposure to panicogenic concentrations of CO_2_.

## References

[1] American Veterinary Medical Association (AVMA) AVMA Guidelines for the Euthanasia of Animals: 2013 Edition. https://www.avma.org/kb/policies/documents/euthanasia.pdf.

[2] European Parliament and the Council of the European Union (2010). Directive 2010/63/eu of the european parliament and of the council of 22 September 2010 on the protection of animals used for scientific purposes. Off. J. Eur. Union.

[3] United States Department of Agriculture (USDA) (2016). Animal Welfare Act and Animal Welfare Regulations.

[4] Canaidan Council on Animal Care (CCAC) (2016). CCAC Guidelines on: Euthanasia of Animals Used in Science.

[5] Office of Laboratory Animal Welfare (OLAW) (2015). Public Health Service Policy on Humane Care and Use of Laboratory Animals.

[6] Tranquilli W., Grimm K., Grimm K., Lamont L., Tranquilli W., Greene S., Robertson S. (2015). Introduction: Use, definitions, history, concepts, classification, and considerations for anesthesia and analgesia. Veterinary Anesthesia and Analgesia: The Fifth Edition of Lumb and Jones.

[7] Peppel P., Anton F. (1993). Responses of rat medullary dorsal horn neurons following intranasal noxious chemical stimulation: Effects of stimulus intensity, duration, and interstimulus interval. J. Neurophysiol..

[8] Danneman P.J., Stein S., Walshaw S.O. (1997). Humane and practical implications of using carbon dioxide mixed with oxygen for anesthesia or euthanasia of rats. Lab. Anim. Sci..

[9] Putnam R.W., Filosa J.A., Ritucci N.A. (2004). Cellular mechanisms involved in CO_2_ and acid signaling in chemosensitive neurons. Am. J. Physiol. Cell Physiol..

[10] Richerson G.B. (2004). Serotonergic neurons as carbon dioxide sensors that maintain ph homeostasis. Nat. Rev. Neurosci..

[11] Guyenet P.G., Stornetta R.L., Abbott S.B., Depuy S.D., Fortuna M.G., Kanbar R. (2010). Central CO_2_ chemoreception and integrated neural mechanisms of cardiovascular and respiratory control. J. Appl. Physiol..

[12] Woods S.W., Charney D.S., Goodman W.K., Heninger G.R. (1988). Carbon dioxide-induced anxiety. Behavioral, physiologic, and biochemical effects of carbon dioxide in patients with panic disorders and healthy subjects. Arch. Gen. Psychiatry.

[13] Masuda A., Sakakibara Y., Ohyabu Y., Yoshino C., Kobayashi T., Honda Y. (2001). Ventilatory vs. Dyspneic responses to augmenting and subsequently declining CO_2_ stimulation in humans. Adv. Exp. Med. Biol..

[14] Banzett R.B., Lansing R.W., Evans K.C., Shea S.A. (1996). Stimulus-response characteristics of CO_2_-induced air hunger in normal subjects. Respir. Physiol..

[15] Shea S.A., Harty H.R., Banzett R.B. (1996). Self-control of level of mechanical ventilation to minimize CO_2_ induced air hunger. Respir. Physiol..

[16] Bailey J.E., Argyropoulos S.V., Kendrick A.H., Nutt D.J. (2005). Behavioral and cardiovascular effects of 7.5% CO_2_ in human volunteers. Depress. Anxiety.

[17] Forsyth J.P., Eifert G.H., Canna M.A. (2000). Evoking analogue subtypes of panic attacks in a nonclinical population using carbon dioxide-enriched air. Behav. Res. Ther..

[18] McKnight P.E., Monfort S.S., Kashdan T.B., Blalock D.V., Calton J.M. (2015). Anxiety symptoms and functional impairment: A systematic review of the correlation between the two measures. Clin. Psychol. Rev..

[19] Fox A.S., Oler J.A., Tromp do P.M., Fudge J.L., Kalin N.H. (2015). Extending the amygdala in theories of threat processing. Trends Neurosci..

[20] Johnson P.L., Federici L.M., Fitz S.D., Renger J.J., Shireman B., Winrow C.J., Bonaventure P., Shekhar A. (2015). Orexin 1 and 2 receptor involvement in CO_2_-induced panic-associated behavior and autonomic responses. Depress. Anxiety.

[21] Akilesh M.R., Kamper M., Li A., Nattie E.E. (1997). Effects of unilateral lesions of retrotrapezoid nucleus on breathing in awake rats. J. Appl. Physiol..

[22] Elam M., Yao T., Thoren P., Svensson T.H. (1981). Hypercapnia and hypoxia: Chemoreceptor-mediated control of locus coeruleus neurons and splanchnic, sympathetic nerves. Brain Res..

[23] Johnson P.L., Samuels B.C., Fitz S.D., Lightman S.L., Lowry C.A., Shekhar A. (2012). Activation of the orexin 1 receptor is a critical component of CO_2_-mediated anxiety and hypertension but not bradycardia. Neuropsychopharmacology.

[24] Walker B.R., Brizzee B.L. (1990). Cardiovascular responses to hypoxia and hypercapnia in barodenervated rats. J. Appl. Physiol..

[25] Cuccheddu T., Floris S., Serra M., Porceddu M.L., Sanna E., Biggio G. (1995). Proconflict effect of carbon dioxide inhalation in rats. Life Sci..

[26] Johnson P.L., Fitz S.D., Hollis J.H., Moratalla R., Lightman S.L., Shekhar A., Lowry C.A. (2011). Induction of c-fos in “panic/defence”-related brain circuits following brief hypercarbic gas exposure. J. Psychopharmacol..

[27] Marotta S.F., Sithichoke N., Garcy A.M., Yu M. (1976). Adrenocortical responses of rats to acute hypoxic and hypercapnic stresses after treatment with aminergic agents. Neuroendocrinology.

[28] Sithichoke N., Malasanos L.J., Marotta S.F. (1978). Cholinergic influences on hypothalamic-pituitary-adrenocortical activity of stressed rats: An approach utilizing choline deficient diets. Acta Endocrinol. (Copenh.).

[29] Sithichoke N., Marotta S.F. (1978). Cholinergic influences on hypothalamic-pituitary-adrenocortical activity of stressed rats: An approach utilizing agonists and antagonists. Acta Endocrinol. (Copenh.).

[30] Forster H.V., Smith C.A. (2010). Contributions of central and peripheral chemoreceptors to the ventilatory response to CO_2_/h+. J. Appl. Physiol..

[31] Fukuda Y., Sato A., Suzuki A., Trzebski A. (1989). Autonomic nerve and cardiovascular responses to changing blood oxygen and carbon dioxide levels in the rat. J. Auton. Nerv. Syst..

[32] Williams R.H., Jensen L.T., Verkhratsky A., Fugger L., Burdakov D. (2007). Control of hypothalamic orexin neurons by acid and CO_2_. Proc. Natl. Acad. Sci. USA.

[33] Nashold B.S., Wilson W.P., Slaughter D.G. (1969). Sensations evoked by stimulation in the midbrain of man. J. Neurosurg..

[34] Wilent W.B., Oh M.Y., Buetefisch C., Bailes J.E., Cantella D., Angle C., Whiting D.M. (2011). Mapping of microstimulation evoked responses and unit activity patterns in the lateral hypothalamic area recorded in awake humans. Technical note. J. Neurosurg..

[35] Samuels B.C., Zaretsky D.V., DiMicco J.A. (2002). Tachycardia evoked by disinhibition of the dorsomedial hypothalamus in rats is mediated through medullary raphe. J. Physiol..

[36] Shekhar A., Katner J.S. (1995). Dorsomedial hypothalamic gaba regulates anxiety in the social interaction test. Pharmacol. Biochem. Behav..

[37] Hess W.R., Brugger M. (1943). Das subkortikake zenrrumder affektriven abwehrreaktion. Helv. Physiol. Acta.

[38] Ziemann A.E., Allen J.E., Dahdaleh N.S., Drebot I, Coryell M.W., Wunsch A.M., Lynch C.M., Faraci F.M., Howard M.A., Welsh M.J. (2009). The amygdala is a chemosensor that detects carbon dioxide and acidosis to elicit fear behavior. Cell.

[39] Feinstein J.S., Buzza C., Hurlemann R., Follmer R.L., Dahdaleh N.S., Coryell W.H., Welsh M.J., Tranel D., Wemmie J.A. (2013). Fear and panic in humans with bilateral amygdala damage. Nat. Neurosci..

[40] Johnson P.L., Hollis J.H., Moratalla R., Lightman S.L., Lowry C.A. (2005). Acute hypercarbic gas exposure reveals functionally distinct subpopulations of serotonergic neurons in rats. J. Psychopharmacol..

[41] Shekhar A., Keim S.R., Simon J.R., McBride W.J. (1996). Dorsomedial hypothalamic gaba dysfunction produces physiological arousal following sodium lactate infusions. Pharmacol. Biochem. Behav..

[42] Mischler S.A., Alexander M., Battles A.H., Raucci J.A., Nalwalk J.W., Hough L.B. (1994). Prolonged antinociception following carbon dioxide anesthesia in the laboratory rat. Brain Res..

[43] Niel L., Weary D.M. (2007). Rats avoid exposure to carbon dioxide and argon. Appl. Anim. Behav. Sci..

[44] Goetz R.R., Klein D.F., Papp L.A., Martinez J.M., Gorman J.M. (2001). Acute panic inventory symptoms during CO_2_ inhalation and room-air hyperventilation among panic disorder patients and normal controls. Depress. Anxiety.

[45] Gorman J.M., Askanazi J., Liebowitz M.R., Fyer A.J., Stein J., Kinney J.M., Klein D.F. (1984). Response to hyperventilation in a group of patients with panic disorder. Am. J. Psychiatry.

[46] Gorman J.M., Fyer M.R., Goetz R., Askanazi J., Liebowitz M.R., Fyer A.J., Kinney J., Klein D.F. (1988). Ventilatory physiology of patients with panic disorder. Arch. Gen. Psychiatry.

[47] Diji A. (1959). Local vasodilator action of carbon dioxide on blood vessels of the hand. J. Appl. Physiol..

[48] Ito T., Moore J.I., Koss M.C. (1989). Topical application of CO_2_ increases skin blood flow. J. Investig. Dermatol..

[49] Hornett T., Haynes A. (1984). Comparison of cardon dioxide/air mixture and nitrogen/air mixture for the euthanasia of rodents. Design of a system for inhalation euthanasia. Anim. Technol..

[50] Oertel B.G., Preibisch C., Martin T., Walter C., Gamer M., Deichmann R., Lotsch J. (2012). Separating brain processing of pain from that of stimulus intensity. Hum. Brain Mapp..

[51] Hummel T., Mohammadian P., Marchl R., Kobal G., Lotsch J. (2003). Pain in the trigeminal system: Irritation of the nasal mucosa using short- and long-lasting stimuli. Int. J. Psychophysiol..

[52] Lee H., Roh S., Kim D.J. (2009). Alcohol-induced blackout. Int. J. Environ. Res. Public Health.

[53] Heath T.S., Burroughs Z., Thompson A.J., Tecklenburg F.W. (2012). Acute intoxication caused by a synthetic cannabinoid in two adolescents. J. Pediatr. Pharmacol. Ther..

[54] Kongara K., McIlhone A.E., Kells N.J., Johnson C.B. (2014). Electroencephalographic evaluation of decapitation of the anaesthetized rat. Lab. Anim..

[55] Van Rijn C.M., Krijnen H., Menting-Hermeling S., Coenen A.M. (2011). Decapitation in rats: Latency to unconsciousness and the “wave of death”. PLoS ONE.

[56] Meyer R.E. (2015). Physiologic measures of animal stress during transitional states of consciousness. Animals.

[57] Moody C.M., Chua B., Weary D.M. (2014). The effect of carbon dioxide flow rate on the euthanasia of laboratory mice. Lab. Anim..

[58] Kelly M.M., Forsyth J.P., Karekla M. (2005). Sex differences in response to a panicogenic challenge procedure: An experimental evaluation of panic vulnerability in a non-clinical sample. Behav. Res. Ther..

[59] Artwohl J., Brown P., Corning B., Stein S., Force A.T. (2006). Report of the aclam task force on rodent euthanasia. J. Am. Assoc. Lab. Anim. Sci..

